# Ozone modified hypothalamic signaling enhancing thermogenesis in the TDP-43^A315T^ transgenic model of Amyotrophic Lateral Sclerosis

**DOI:** 10.1038/s41598-022-25033-4

**Published:** 2022-12-02

**Authors:** Sara Rodríguez-Sánchez, Nicolas Valiente, Susana Seseña, Marta Cabrera-Pinto, Ana Rodríguez, Alfonso Aranda, Llanos Palop, Carmen M. Fernández-Martos

**Affiliations:** 1 Faculty of Environmental Sciences and Biochemistry, University of Castilla-La Mancha, Toledo, Spain; 2 Division of Terrestrial Ecosystem Research, Centre for Microbiology and Environmental Systems Science, University of Vienna, Vienna, Austria; 3 Hospital Nacional de Parapléjicos, SESCAM, Toledo, Spain; 4 Faculty of Chemical Science and Technology, University of Castilla-La Mancha, Ciudad Real, Spain; 5 Wicking Dementia Research and Education Centre, College of Health and Medicine, University of Tasmania, Hobart, Tasmania Australia

**Keywords:** Molecular biology, Neuroscience

## Abstract

Amyotrophic lateral sclerosis (ALS), a devastating progressive neurodegenerative disease, has no effective treatment. Recent evidence supports a strong metabolic component in ALS pathogenesis. Indeed, metabolic abnormalities in ALS correlate to disease susceptibility and progression, raising additional therapeutic targets against ALS. Ozone (O_3_), a natural bioactive molecule, has been shown to elicit beneficial effects to reduce metabolic disturbances and improved motor behavior in TDP-43^A315T^ mice. However, it is fundamental to determine the mechanism through which O_3_ acts in ALS. To characterize the association between O_3_ exposure and disease-associated weight loss in ALS, we assessed the mRNA and protein expression profile of molecular pathways with a main role in the regulation of the metabolic homeostasis on the hypothalamus and the brown adipose tissue (BAT) at the disease end-stage, in TDP-43^A315T^ mice compared to age-matched WT littermates. In addition, the impact of O_3_ exposure on the faecal bacterial community diversity, by Illumina sequencing, and on the neuromuscular junctions (NMJs), by confocal imaging, were analysed. Our findings suggest the effectiveness of O_3_ exposure to induce metabolic effects in the hypothalamus and BAT of TDP-43^A315T^ mice and could be a new complementary non-pharmacological approach for ALS therapy.

## Introduction

Amyotrophic Lateral Sclerosis (ALS) is considered the third most common neurodegenerative disease worldwide^1^, and is becoming a disease with a significant impact and much community awareness for many countries across the globe. Over 60% of patients die within 3–5 years of diagnosis. There is no cure for ALS. Although much effort has been made in the past two decades to understand the complexity and heterogeneity of ALS, the mechanisms by which progressive degeneration and death of motor neurons occur have not yet been elucidated. Indeed, both genetic and risk factors from gene-environment interactions, including metabolic alterations, contribute to the disease progression and pathogenesis.

Not until a couple of years ago that both clinical and epidemiological studies provided for the first time evidence that ozone (O_3_), a highly oxidative gas, may be a potential novel therapeutic strategy in the treatment of degenerative disorders^2–5^. In this context, clinical trials evidenced the effectiveness of O_3_ therapy in the treatment of multiple sclerosis^2,3,5^, due to its potential to induce controlled oxidative stress^6^, through a mechanism of action involving its interaction with the nuclear factor erythroid-derived 2–like 2 (Nrf2)^3^, a key regulator of inducible antioxidant responses^7^. Additionally, clinical data indicated the immunomodulatory effect of O_3_ therapy^8^, and animal work demonstrated the effectiveness of this gas to activate the hypothalamus–pituitary-adrenal (HPA) axis^9^, which has the potential to alter the composition of the gut microbiota and increase gastrointestinal permeability^10,11^, suggesting the potential role of O_3_ to induce controlled metabolic effects.

We previously reported that repeated exposure to O_3_ significantly reduce metabolic disturbances in the mouse model of ALS based on TDP-43 proteinopathy (TDP-43^A315T^ mice)^12^. However, the underlying molecular changes that link O_3_ exposures to disease-associated weight loss in this transgenic (Tg) line were not determined. In the current study, we have examined the relationships between O_3_ exposure and induced metabolic effects in TDP-43^A315T^ mice, by mean of the study of the molecular pathways with a main role in the regulation of the metabolic homeostasis in TDP-43^A315T^ mice. To our knowledge, this work represents the first insight regarding the potential use of this gas as a potential complementary approach to improve metabolic and endocrine response in ALS.

## Methods

### Animals

Cohorts of male Tg Prp-TDP43^A315T^ mice (Strain No. 010700, United States)^12^ and age-matched WT littermates (non-Tg C57Bl6/J mice) were used in this study. This Tg mouse model of ALS^13^ expresses human transactive response DNA-binding protein 43 (TDP-43) with an A315T mutation (hTDP-43^A315T^)^12^. Animals expressing the hTDP-43 transgene were confirmed via PCR following the protocol described^14^. Animals were group-housed under standard housing conditions with a 12 h light–dark cycle, and food and water ad libitum. The maintenance and use of mice and all experimental procedures were approved by the Animal Ethics Committee of the Hospital Nacional de Parapléjicos (Approval no. 26/OH 2018) accordingly with the Spanish Guidelines for the Care and Use of Animals for Scientific Purposes in compliance with ARRIVE guidelines (https://arriveguidelines.org). All analyses were conducted by personnel blinded to the animal genotype.

### Experimental design and O_3_ exposure

Mice at 42 days of age (asymptomatic stage of disease) underwent two consecutive weeks of O_3_ vs. FA (Filtered Air) exposure following the protocol described^13,15^. Each genotype of mice was divided into two subgroups (n = 3- 7 TDP-43^A315T^ mice/subgroup and n = 5–8 WT mice/subgroup) according to the exposure. O_3_ was generated from pure O_2_ with a BTM 802 N generator and distributed in a Plexiglas chamber (50 × 35 × 35 cm) together with zero air at a total flow of 15 L/min. The O_3_ concentration of the ambient air in the chamber was kept constant at 0.25 ppm and was continuously monitored by an Environment O342M analyser (Envea, France). This concentration is higher than both EPA National Ambient Air Quality Standard (NAAQS) (0.075 ppm) and the recommended OMS guideline value (0.05 ppm), but mice are routinely recognized as less susceptible to O_3_ exposure than humans due to obligate nose breathing and other intrinsic factors^16^. FA was obtained by filtering regular air through activated charcoal to reduce O_3_ concentration to a minimum of (< 0.02 ppm). During the 15 consecutive days of exposure (4 h/day), mice had food and water ad libitum, and their general health were monitored closely in terms of their mobility or level of activity. No difference in body weight gain between groups (O_3_ group vs*.* FA group) was observed. Then, to monitor disease onset (defined as the last day of individual peak body weight before a gradual loss occurs) all mice were weighed and assessed three times per week, after which they were checked daily until the end-stage of disease, defined as when weight is 20% below the initial weight on three consecutive days (~ 95–100 ± 2 days). Then the mice were euthanized.

### Fecal collection, DNA extraction and Illumina sequencing and analysis

Two to three freshly evacuated fecal pellets were collected from each mouse in the morning, prior to the end-stage of disease. Briefly, mice temporarily were placed individually into empty autoclaved cages and allowed to defecate. Fecal pellets were collected aseptically from each mouse and placed into a sterile tube. Faecal samples were stored at − 80 °C prior to DNA extraction.

Genomic DNA was extracted with a Purelink Microbiome DNA purification kit (Invitrogen) according to the manufacturer’s instructions, and subjected to high throughput sequencing. DNA yields were determined through fluorometry (Qubit, Life Technologies, Carlsbad, CA) by using a reagent kit (Quant-iT BR dsDNA Kit, Invitrogen, Carlsbad, CA) according to the manufacturer’s instructions. Amplicons purification and Illumina sequencing were performed commercially by Macrogen, Inc., Korea (www.macrogen.com).

A total of 3,484,598 reads were obtained with an average GC content of (54.5 ± 0.6)%, whereas the ratio of bases that have pared quality score of over 20 (Q20) and 30 (Q30) were (93.4 ± 0.5)% and (86.1 ± 0.7)%, respectively. Raw sequences were processed using CUTADAPT^17^ to remove primers. Then, forward and reverse reads were trimmed at 250 and 210 bp, respectively, using DADA2^18^. After being denoised and dereplicated, the remaining forward and reverse reads were assembled. Chimeras were removed using the ‘removeBiomeraDenova’ function in the DADA2 pipeline, which resulted in the final taxon table based on 1,053,851 reads. Taxonomic annotation was performed using the SILVA 132 database^19^. Analyses and graphical representations were performed in R version 4.1.0 (R Core Team., 2013)^20^ using vegan^21^, phyloseq^22^, Microbiome^23^, DESeq2^24^, MASS^25^, ggplot2^26^, ampvis2^27^ and VennDiagram^28^ packages. Sample metadata, taxonomic units as amplicon sequencing variants (ASV) matrix and taxa information were imported into a phyloseq object. Then, alpha diversity measures were calculated (i.e. ASV observed, Chao1, Shannon, Gini-Simpson and Pielou’s Eveness indexes). Bray–Curtis distances were used for calculating beta diversity and non-metric multidimensional scaling (NMDS). Permutational multivariate analysis of variance (PerMANOVA) was carried out based on the Bray–Curtis dissimilarity matrix by using permutest and adonis functions from vegan package.

### Tissue collection and sample preparation

Animals were euthanized with sodium pentobarbitone (140 mg/kg) injected intraperitoneally, and transcardially perfused with 0.01 M phosphate buffered saline (PBS; pH 7.4). For molecular biology experiments, tissue samples of hypothalamus and brown adipose tissue (BAT) were processed independently to extract both mRNA and proteins, for real time PCR or Western-blotting analysis following the protocol described^29^. Hypothalamus and BAT were immediately frozen on dry ice and stored at − 80 °C for later analysis. For IHC analysis, TA samples were dissected, post-fixed overnight in 4% paraformaldehyde (PFA in 0.01 M PBS), and then transferred to 18% and then 30% sucrose solutions overnight^30^. Serial cryosections (50 µm thick) were cut on a cryostat (Leica CM 1850).

### RNA isolation and RT-qPCR

Total RNA was isolated from hypothalamus and BAT, respectively, using the RNeasy Lipid Tissue Mini Kit (Qiagen, Hilden, Germany) according to the manufacturer’s instructions. Complementary DNA (cDNA) (0.5 µg of total RNA for hypothalamus and 1 µg for BAT, respectively) and the relative quantification of each gene was performed as described previously^31^. The 18S rRNA was used as a control to normalize gene expression^32^. The reactions were run on an ABI PRISM 7900 Fast Sequence Detection System instrument and software (Applied Biosystem) according to the manufacturer's protocol. Primers were designed using NCBI/Primer-BLAST software (Tables 1, 2). Relative quantification for each gene was performed by the ∆∆Ct method^33^.

**Table 1:** Primers used for quantitative real time reverse transcription-polymerase chain reaction (RT-qPCR) of genes implicated in regulation of energy homeostasis in the hypothalamus.

Gene name	Forward primer	Reverse primer
*POMC*	5′-AGAACGCCATCATCAAGAAC-3′	5′-AAGAGGCTAGAGGTCATCAG-3′
*AgRP*	5′-TGGCCTCAAGAAGACAACTG-3′	5′-CATTGGCTAGGTGCGACTAC-3′

**Table 2:** Primers used for RT-qPCR of genes involved in the metabolism of BAT.

Gene name	Forward primer	Reverse primer
*Prdm16*	5′-ATGGGCTTTGACCATACCCG-3′	5′-TCGGCTCCAAAGCTAACAGG-3′
*PGC1α*	5′-GCACGCAGCCCTATTCATTG-3′	5′-TGAGTCTCGACACGGAGAGT-3′
*PPARγ*	5′-TGAAAGAAGCGGTGAACCACTG-3′	5′-TGGCATCTCTGTGTCAACCATG-3′
*AdipoQ*	5′-TCCCAATGTACCCATTCGCT-3′	5′-AGAGTCCCGGAATGTTGCAG-3′
*Fabp4*	5′-CCTTCAAACTGGGCGTGGAA-3′	5′-CCCCGCCATCTAGGGTTATG-3′
*GLUT4*	5′-GCTCTGACCGATGGGGAACC-3′	5′-AAACTGAAGGGAGCCAAGCA-3′
*UCP1*	5′-AGTACCCAAGCGTACCAAGC-3′	5′-GACCCGAGTCGCAGAAAGA-3′
*Leptin*	5′-TTCACACACGCAGTCGGTAT-3′	5′-GAAGTCCAAGCCAGTGACCC-3′
*Ob-Rb*	5′-CCCCCACTGAAAGACAGCTT-3′	5′-TTCACATCCCCGAAGACTGC-3′

### Protein extraction and western blot analysis

Proteins from the hypothalamus were extracted using RIPA buffer (Sigma Aldrich) containing a cocktail of protease inhibitors (Roche) as described previously^29^. Denatured protein samples (30 µg) from each group were electrophoresed into Bolt® Bis–Tris Plus gels (Invitrogen), transferred to PVDF membranes (BioRad) and incubated with primary antibodies [rabbit anti-long-form of the leptin receptor (Ob-Rb) (1:500; Abcam), mouse anti-Akt (1:250; Santa Cruz), rabbit anti-phospho Akt (Ser473) (1:500; Cell Signaling), mouse anti-STAT3 (1:250; Santa Cruz Biotechnology), rabbit anti-phospho STAT3 (Tyr705) (1:500; Santa Cruz Biotechnology), rabbit anti-supressor of cytokine signaling 3 (SOCS-3) (1:500; Cell Signaling), and rabbit anti-neuropeptide Y (NPY) (1:200; Abcam)] overnight. Subsequently, a corresponding anti-rabbit or anti-mouse horseradish peroxidase (HRP)-conjugated secondary antibody (Vector Laboratories) were used as described previously^29^. Mouse anti-GAPDH (1:5000, Millipore) and/or mouse anti-actin (1:1000; Cell Signaling) were used as a loading control and band intensity was measured as the integrated intensity using ImageJ software (v1.4; NIH). All data were normalized to control values on each membrane.

### Muscle histology and analysis of NMJs

Tibialis anterior (TA) samples were processed as is previously described. The stain of NMJs were conducted following the protocol described^50^, with slight modifications. Briefly, slides were brought up to RT and incubated for 1 h with alpha(α)-bungarotoxin (α-BTX) (Invitrogen) at 1:500 in TBS. Then, slides were incubated in blocking solution (Dako) for 1 h. Primary antibodies against beta(β)-III-tubulin (Promega) and synaptophysin (SYN) (GeneTex) were applied at 1:150 and 1:250, respectively, in blocking solution, and incubated at 4 °C overnight. Sections were then incubated with their corresponding mouse Alexa488 and rabbit Alexa647-conjugated secondary antibodies (Thermo Fisher Scientific), both diluted 1:1000 in blocking solution, for 1 h at room temperature (RT). Finally, the slides were coverslipped in “Immumount” (Thermo Scientific) and confocal images were collected at RT with × 20 objective, and LAS-AF software (Leica).

NMJs from flattened z stacks of TA were analysed (ImageJ software v1.4; NIH) by personnel blinded to the animal genotype. Brightness and contrast thresholds were set to optimize the signal-to-noise ratio of the presynaptic staining (β-III-tubulin and SYN). Innervated NMJs were defined as having observed overlap of staining for pre- and post-synaptic elements. Partial denervated NMJs were defined as α-BTX signal in the presence of incomplete presynaptic staining. Denervated NMJs were defined as α-BTX signal in the absence of presynaptic staining^50–53^.

### Statistical analysis

For molecular biology experiments and IHC analysis, respectively, two-way ANOVA was used followed by Dunett’s post hoc test, to compare all groups with control WT-FA mice, while Tukey’s post hoc test were used for multiple comparisons between all groups. Statistical analysis was performed using GraphPad Prism software (version 8.3.1) with *p* < 0.05 (CI 95%) considered significant. Values were reported as means ± standard error of the mean (SEM).

## Results

### O_3_ exposure significantly altered the expression levels of Akt pathway in the hypothalamus of TDP-43^A315T^ mice

**Figure 1:**
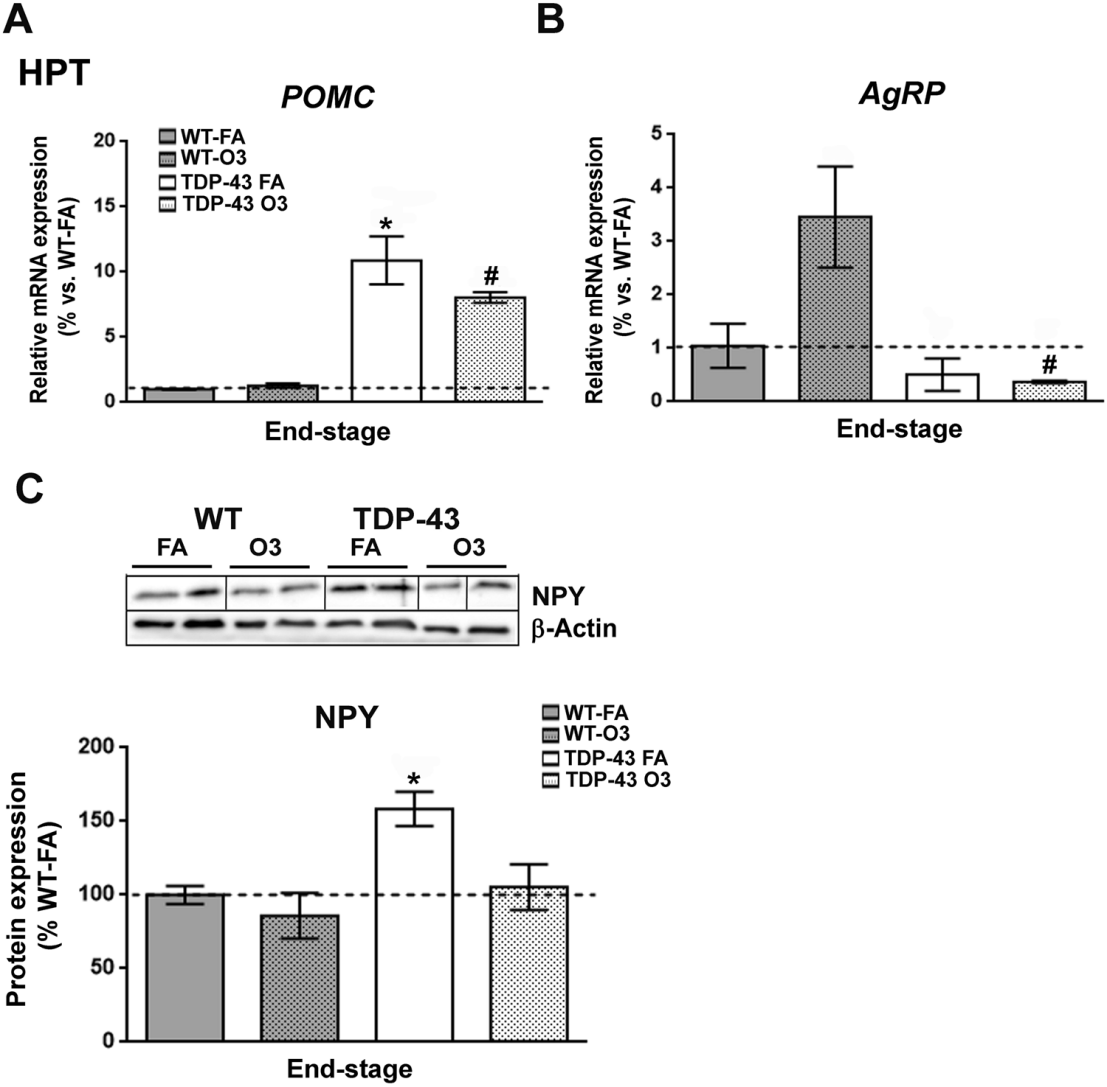
**Alterations in anorexigenic and orexigenic neuropeptides in the hypothalamus of TDP-43**^**A315T**^ **mice in response to O**_**3**_**.** (**A**) mRNA expression of *POMC* and (**B**) *AgRP* neuropeptides was assessed by RT-qPCR in the hypothalamus of TDP-43^A315T^ mice exposed to FA (n = 3 mice) or O_3_ (n = 4 mice) compared to WT controls exposed to FA (n = 5 mice) or O_3_ (n = 5 mice), at the end-stage of disease. (**C**) Representative β-Actin-normalized immunoblot images and quantification of NPY protein in the hypothalamus of TDP-43^A315T^ mice compared to age-matched WT littermate controls. The white lines in the immunoblot images represent the lanes that were run on the same gel but were non-contiguous. Values are expressed as the mean ± SEM for the different groups. Comparison between groups was performed by two-way ANOVA followed by Dunnett’s post hoc test to compare all groups with WT-FA, while Tukey’s post hoc test was used for multiple comparisons between all groups, where * *p* < 0.05 vs. WT mice FA-exposed; # *p* < 0.05 vs. WT mice O_3_-exposed; ** *p* < 0.05 vs. TDP-43^A315T^ FA-exposed. In the immunoblot images, representative bands were run on the same gel but were non-continuous. Abbreviations: WT, Wild-type mice; TDP-43,TDP-43^A315T^ mice; FA*,* filtered air; O_3_, ozone; HPT*,* hypothalamus.

Since metabolic abnormalities have been reported in both ALS patients^34^ and mouse models of ALS^29,35–37^, and as we previously reported a progressive decline in body weight in TDP-43^A315T^ mice^13^, we evaluated if genes involved in metabolism were affected in the hypothalamus of TDP-43^A315T^ mice compared to WT mice in responses to FA or O_3_ exposure (Fig. 1). RT-qPCR analysis demonstrated that there was a significant effect of genotype on the expression profile of *POMC* (*p* < 0.0001) and *AgRP* (*p* = 0.01) transcripts in the hypothalamus (Fig. 1A,B). Tukey’s post hoc test demonstrated a significant increase in *POMC* mRNA expression in both TDP-43^A315T^ mice FA and O_3_-exposed compared to WT controls in response to FA and O3 (*p* = 0.0003 and *p* = 0.003, respectively; Fig. 1A). Conversely, Tukey’s post hoc test demonstrated a significant downregulation of *AgRP* mRNA levels in the hypothalamus of TDP-43^A315T^ mice O_3_-exposed compared to WT controls in response to O_3_ (*p* = 0.01; Fig. 1B). Additionally, immunoblotting analysis demonstrated that there was a significant effect of genotype on the protein expression levels profile of NPY (*p* = 0.01) in the hypothalamus (Fig. 1C; Supplementary Fig. S1). Tukey’s post hoc test demonstrated a significant hypothalamic upregulation of NPY protein levels in TDP-43^A315T^ mice FA-exposed relative to WT controls in response to FA (*p* = 0.04; Fig. 1C; Supplementary Fig. S1).

**Figure 2:**
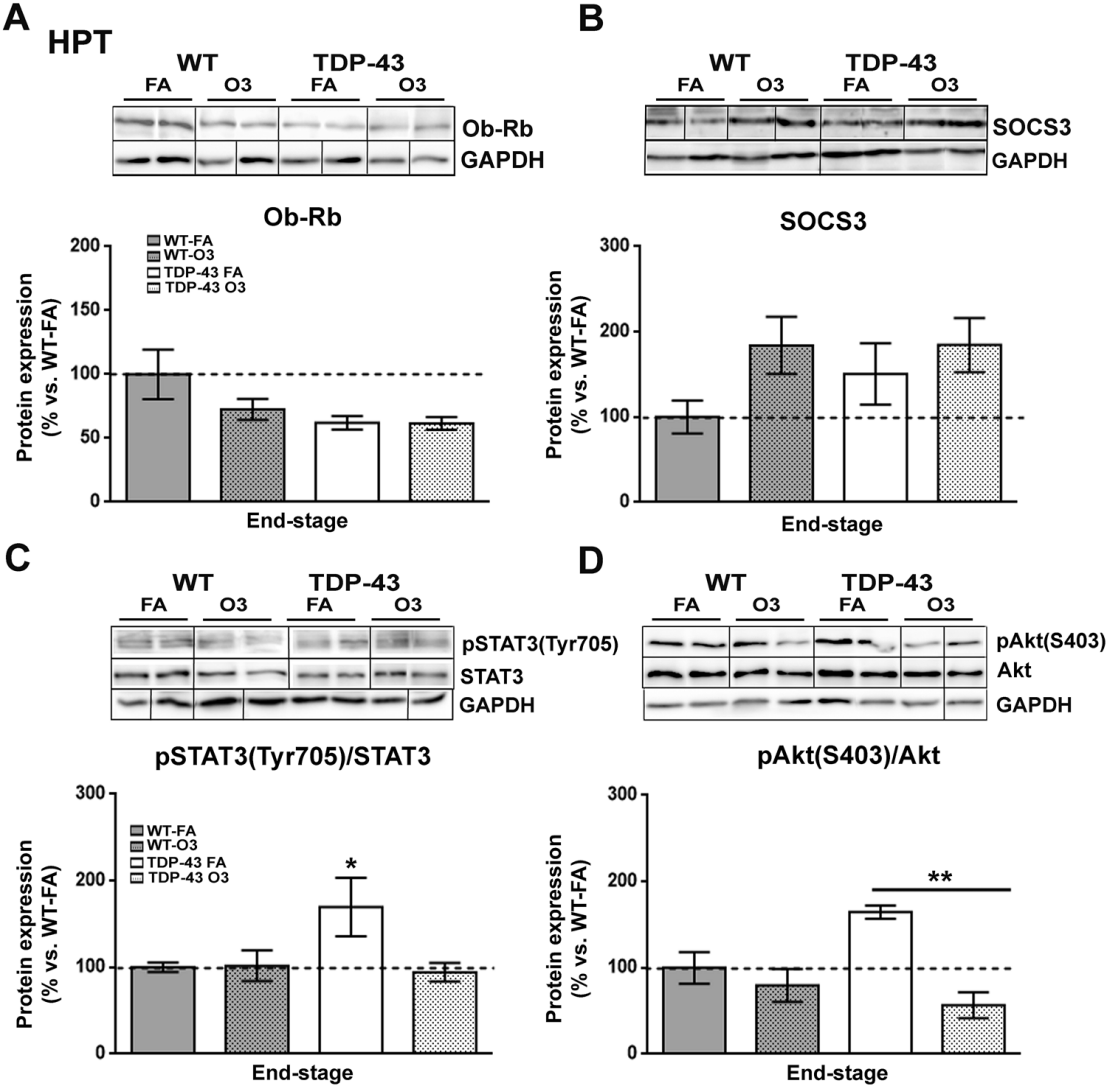
**Alterations in serine phosphorylation of Akt in the hypothalamus of TDP-43**^**A315T**^ **mice in response to O**_**3**_** exposure.** (**A**) Representative β-Actin-normalized immunoblot images and quantitation of Ob-Rb receptor, (**B**) SOCS3, (**C**) pSTAT3 (pTyr705-STAT3) protein, (**D**) pAkt (pSer473-Akt) protein, respectively, in the hypothalamus of TDP-43^A315T^ mice exposed to FA (n = 3 mice) or O_3_ (n = 4 mice) compared to WT controls exposed to FA (n = 5 mice) or O_3_ (n = 5 mice) at the end-stage of disease. The white lines in the immunoblot images represent the lanes that were run on the same gel but were non-contiguous. Values are expressed as the mean ± SEM for the different groups. Comparison between groups was performed by two-way ANOVA followed by Dunnett’s post hoc test to compare all groups with WT-FA, while Tukey’s post hoc test was used for multiple comparisons between all groups, where * *p* < 0.05 vs. WT mice FA-exposed; # *p* < 0.05 vs. WT mice O_3_-exposed; ** *p* < 0.05 vs. TDP-43^A315T^ FA-exposed. In the immunoblot images, representative bands were run on the same gel but were non-continuous. Abbreviations: HPT, hypothalamus; Ob-Rb, long form of leptin receptor; SOCS3, suppressor of cytokine signaling 3; STAT3, signal transducer and activator of transcription 3; Akt*,* serine/threonine kinase.

We next investigated the protein levels of Ob-Rb, the long isoform of leptin receptor, SOCS3, a main inhibitor of the leptin signaling, and the phosphorylation levels of Akt (pSer^473^-Akt) and STAT3 (pTyr^705^-STAT3) proteins (Fig. 2), as they are important targets in the regulation of glucose and energy metabolism^38^. Immunoblotting analysis demonstrated no significant effect of either genotype or O_3_ exposure on Ob-Rb and SOCS3 protein expression levels (Fig. 2A,B; Supplementary Fig. S2). With respect of leptin signaling pathways, immunoblotting analysis demonstrated a significant increase on the phosphorylation levels of STAT3 protein in the hypothalamus of TDP-43^A315T^ mice FA-exposed compared to WT controls in response to FA (*p* = 0.04) (Fig. 2C; Supplementary Fig. S2). However, there was a significant effect of the O_3_ exposure (*p* = 0.0035) on the phosphorylation levels of Akt protein in the hypothalamus of TDP-43^A315T^ mice (Fig. 2D). Tukey’s post hoc test demonstrated a statistically significant hypothalamic decrease on the phosphorylation levels of Akt protein in TDP-43^A315T^ mice O_3_-exposed compared to TDP-43^A315T^ mice (*p* = 0.008) in responses to FA (Fig. 2D; Supplementary Fig. S2).

### O_3_ exposure decreased the expression profile of genes involved in metabolism and thermogenesis in the BAT of TDP-43^A315T^ mice

**Figure 3:**
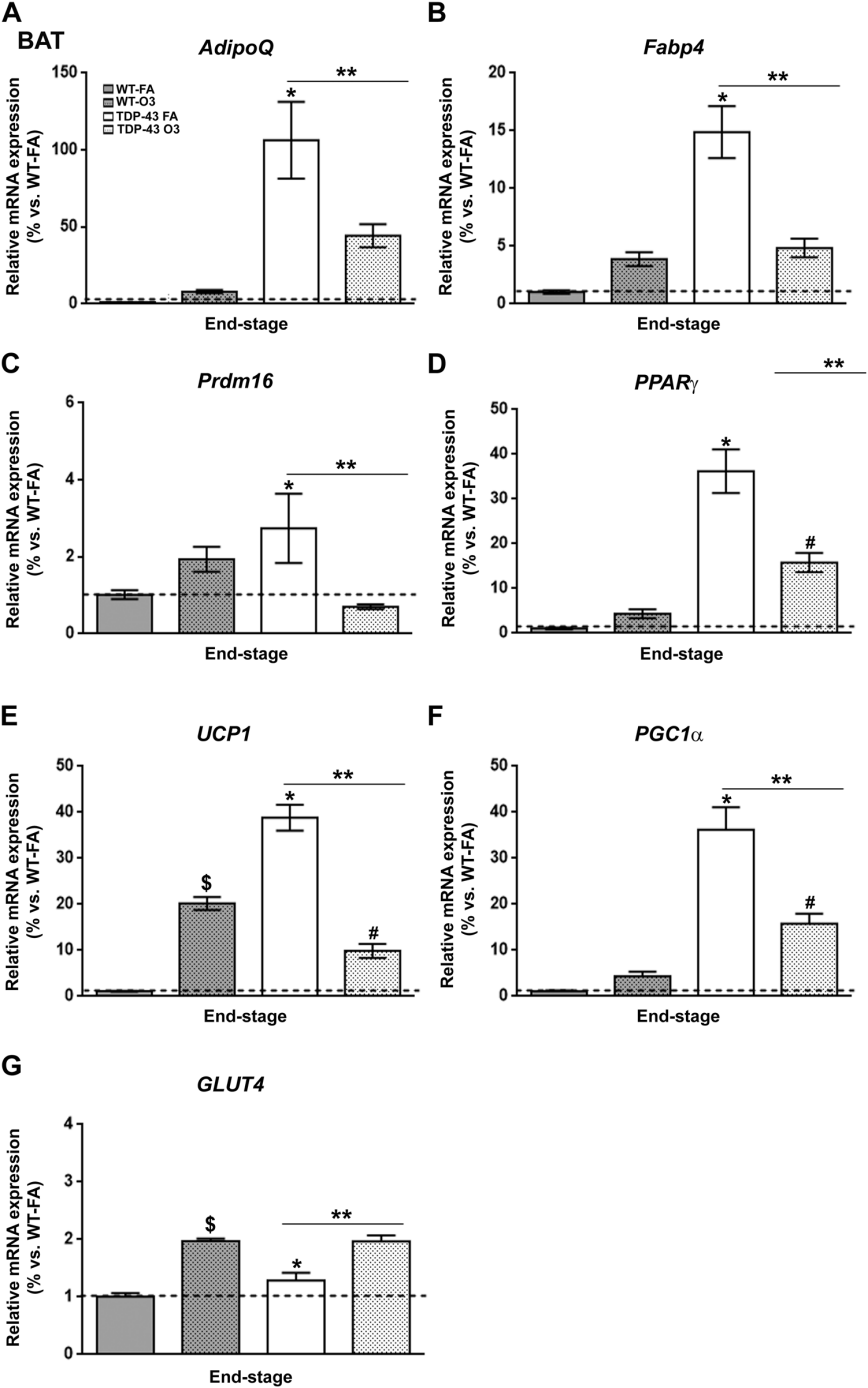
**Alterations in the expression of genes involved in brown adipocyte differentiation in the BAT of TDP-43**^**A315T**^ **mice in response to O**_**3**_** exposure.** (**A**) *AdipoQ*, (**B**) *Fabp4*, (**C**) *Prdm16*, (**D**) *PPARγ*, (**E**) *UCP1*, (**F**) *PGC1α* and (**G**) *GLUT4* mRNA expression was assessed by RT-qPCR in the BAT of TDP-43^A315T^ mice exposed to FA (n = 5 mice) or O_3_ (n = 7 mice) compared to WT controls exposed to FA (n = 6 mice) or O_3_ (n = 8 mice) at the end-stage of disease. Values are expressed as the mean ± SEM for the different groups (n = 3–8 per genotype/exposure). Comparison between groups was performed by two-way ANOVA followed by Dunnett’s post hoc test to compare all groups with WT-FA, while Tukey’s post hoc test was used for multiple comparisons between all groups, where ^$ ^*p* < 0.05 WT O_3_-exposed vs. WT mice FA-exposed; * *p* < 0.05 vs. WT mice FA-exposed; # *p* < 0.05 vs. WT mice O_3_-exposed; ** *p* < 0.05 vs. TDP-43^A315T^ FA-exposed. Abbreviations: WT, Wild-type mice; TDP-43, TDP-43^A315T^ mice; FA, filtered ai; O_*3*_,  ozone; BAT, brown adipose tissue.

BAT is a thermogenic organ with an important role in controlling energy expenditure and the regulation of body weight^39^. Interestingly, animal work has shown that acute O_3_ exposure causes endocrine and metabolic changes, increasing food intake and body fat mass^40^. In this context, to determine the metabolic impact of O_3_ exposure on altering BAT thermogenic activity in TDP-43^A315T^ mice compared to WT mice in responses to FA or O_3_ exposure, we first conducted RT-qPCR analysis to examine the expression profile of genes involved in brown adipocyte differentiation (Fig. 3). Our results indicated a significant effect of genotype and exposure (*p* < 0.05) on the expression profile of *AdipoQ*, *Fabp4*, *Prdm16* and *PPARγ* transcripts in BAT across groups (Fig. 3A–D). In TDP-43^A315T^ mice FA-exposed, *AdipoQ*, *Fabp4*, *Prdm16* and *PPARγ* mRNA levels were significantly upregulated compared to WT controls in responses to FA (*p* = 0.0002, *p* < 0.0001, *p* = 0.04 and *p* < 0.0001, respectively). Additionally, Tukey’s post hoc test demonstrated an overall statistically significant downregulation of all genes (*AdipoQ: p* = 0.01, Fig. 3A; *Fabp4*: *p* < 0.0001, Fig. 3B; *Prdm16: p* = 0.01, Fig. 3C; and *PPARγ*: *p* = 0.0001, Fig. 3D) in the BAT of TDP-43^A315T^ mice O_3_-exposed compared to TDP-43^A315T^ mice in responses to FA.

We next examined the expression of genes involved in BAT-mediated thermogenesis, including *UCP1* and *PGC1α* (Fig. 3E,F). There were significant effects of genotype (*p* < 0.0001) and exposure (*p* = 0.008 and *p* = 0.009, respectively) on the expression profile of both genes in the BAT. In TDP-43^A315T^ mice FA-exposed, *UCP1* and *PGC1α* mRNA levels were significantly upregulated compared to WT controls in responses to FA (*p* = 0.003 and *p* = 0.0001, respectively), while a different pattern of expression was observed in TDP-43^A315T^ mice O_3_-exposed compared to WT controls in responses to O_3_ (Fig. 3E,F). Additionally, RT-qPCR analysis demonstrated that O_3_ exposure modified *UCP1* and *PGC1α* mRNA levels in TDP-43^A315T^ mice, with a statistically significant downregulation on their expression compared to TDP-43^A315T^ mice in responses to FA (*p* < 0.0001, Fig. 3E,F).

**Figure 4:**
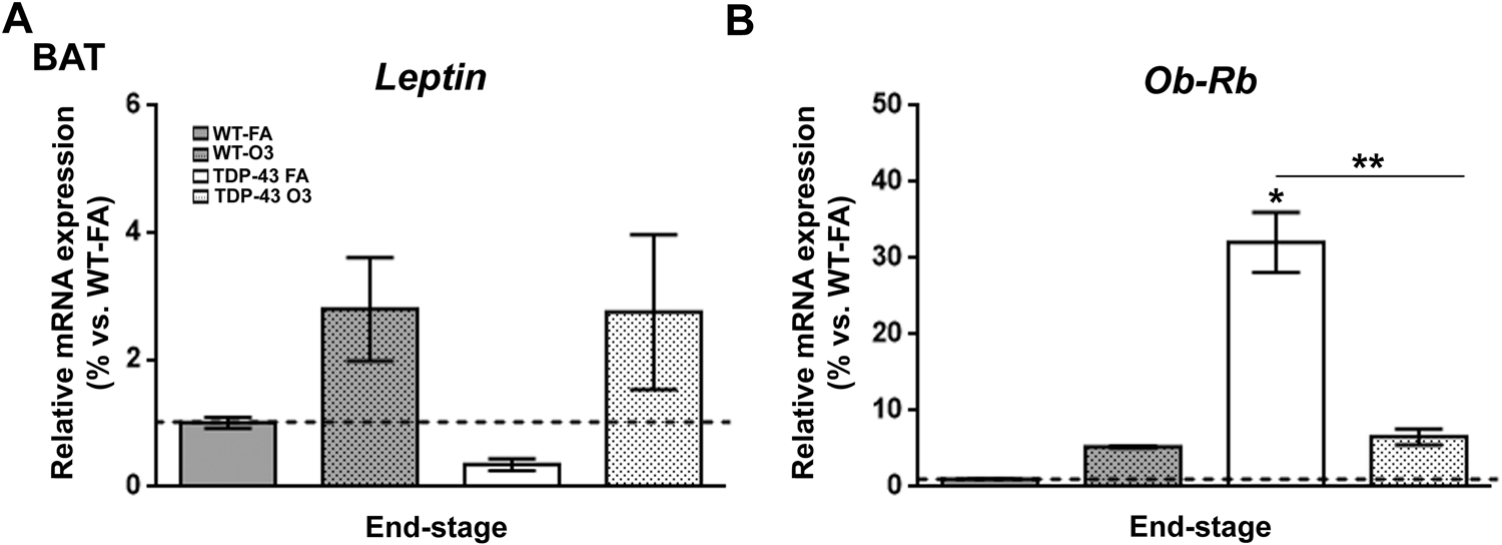
**Alterations in Ob-Rb in the BAT of TDP-43**^**A315T**^** mice in response to O**_**3**_ **exposure**. (**A**) *Leptin* and (**B**) *Ob-Rb* mRNA expression was assessed by RT-qPCR in the BAT of TDP-43^A315T^ mice exposed to FA (n = 5 mice) or O_3_ (n = 7 mice) compared to WT controls exposed to FA (n = 6 mice) or O_3_ (n = 8 mice) at the end-stage of disease. Values are expressed as the mean ± SEM for the different groups. Comparison between groups was performed by two-way ANOVA followed by Dunnett´s post hoc test to compare all groups with WT-FA, while Tukey’s post hoc test was used for multiple comparisons between all groups, where * *p* < 0.05 vs. WT mice FA-exposed; # *p* < 0.05 vs. WT mice O_3_-exposed; ** *p* < 0.05 vs. TDP-43^A315T^ FA-exposed. Abbreviations: WT, Wwild-type mice; TDP-43, TDP-43^A315T^ mice; FA, filtered air; O_3_ , ozone; BAT, brown adipose tissue.

Finally, to further examine the effect of O_3_ exposure on BAT, we analysed the expression profile of genes known to mediate glucose uptake, particularly *GLUT4*, which is significantly expressed in BAT^41^ (Fig. 3G). Remarkably, as leptin modulates body weight gain and energy expenditure^42,43^, at least in part, by modulating thermogenesis^44^, and, as it has been provided that leptin treatment increase body temperature in leptin deficient mice (*ob/ob* mice)^45^, we also asked how *Leptin* and *Ob-Rb* transcripts were affected in the BAT of TDP-43^A315T^ mice in response to O_3_ exposure (Fig. 4). RT-qPCR analysis demonstrated a statistically significant upregulation of *GLUTt4* mRNA levels in the BAT of TDP-43^A315T^ mice in responses to O_3_ exposure (*p* < 0.0015, Fig. 3G). There was no effect of genotype on the expression profile of *Leptin* transcript in the BAT (Fig. 4A). In contrast, RT-qPCR analysis demonstrated a statistically significant BAT upregulation of *Ob-Rb* mRNA levels in TDP-43^A315T^ mice FA-exposed compared to WT controls in responses to FA exposure (*p* < 0.0001, Fig. 4B). Tukey’s post hoc test demonstrated a statistically significant downregulation of *Ob-Rb* mRNA levels (*p* < 0.0001, Fig. 4B) in the BAT of O_3_-exposed TDP-43^A315T^ mice compared to TDP-43^A315T^ mice in responses to FA.

### O_3_ exposure did not induce fecal microbiota compositional changes in TDP-43^A315T^ mice

**Figure 5:**
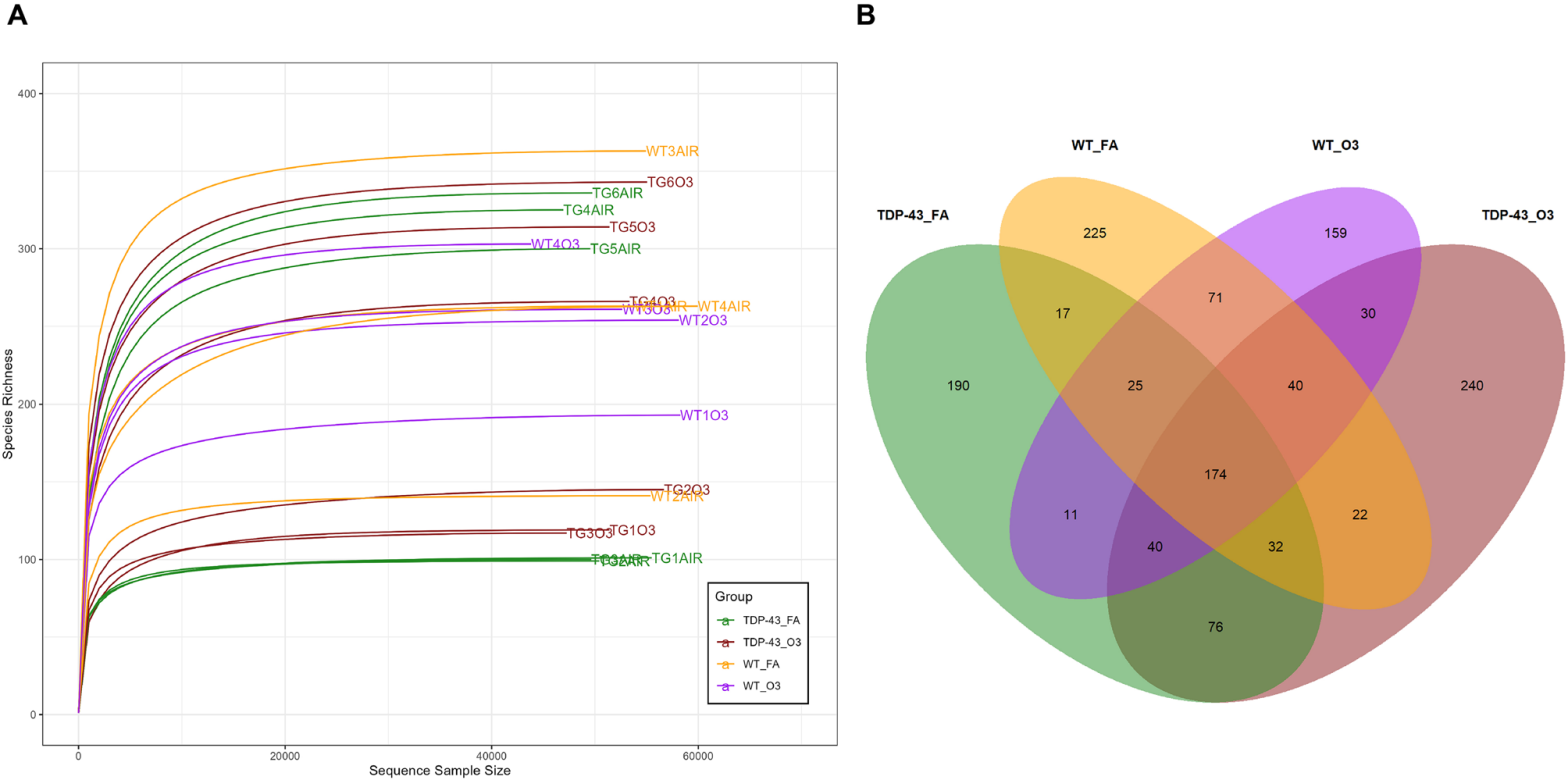
**WT controls and TDP-43**^**A315T**^ **mice fecal microbiota diversity.** (**A**) Rarefaction curves for the samples analysed within the four groups (TDP-43-FA, TDP-43-O_3_, WT-FA and WT-O_3_). (**B**) Venn diagram of four groups (TDP-43-FA, TDP-43-O_3_, WT-FA and WT-O_3_) and their intersections. The numbers correspond to the number of ASVs in each subset and intersection. Abbreviations: WT, Wild-type mice; TDP-43*,* TDP-43^A315T^ mice; FA, filtered air; O_3_, ozone.

**Figure 6:**
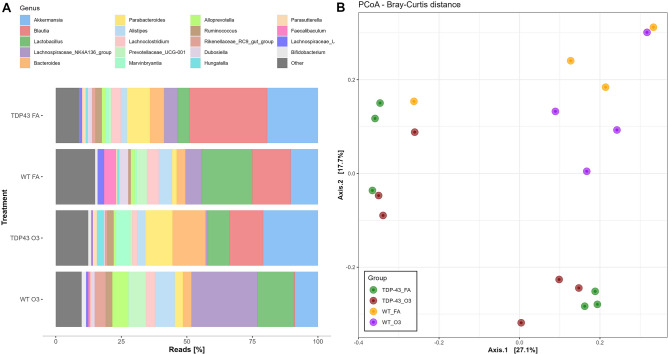
**TDP-43 **^**A315T**^ **mice develop gut microbiome compositional changes. **(**A**) Relative abundance at genus level obtained by 16S rRNA amplicon sequencing. (**B**) Bray–Curtis PCoA at the end-stage of disease. Abbreviations: WT*,* Wild-type mice; TG*,* TDP-43^A315T^ mice; FA, filtered air; O_3,_ ozone.

As it has been reported that the HPA axis closely interact with the gut microbiota^46^, we next compared fecal gut microbiome composition in TDP-43^A315T^ mice and age-matched WT littermate controls, in responses to FA or O_3_ exposure. Microbial-mediated effects on ALS neuropathology were assessed through 16S rRNA amplicon sequencing in fecal samples from TDP-43^A315T^ and WT controls prior to the end-stage of disease. The rarefaction curve suggested that ASV richness is not affected by either genotype or exposure, since any trend is observed as indicated by the mix higher curves under the same sequencing depth (Fig. 5A). Additionally, all the curves tend to be smooth and reach the plateau, which indicates that the amount of sequencing data was sufficient and reasonable, and that more data would only produce a few new ASVs. The total number of observed genera (alpha diversity) was similar in both TDP-43^A315T^ mice and WT controls whether they were exposed to FA or O_3_. However, evenness was higher in WT mice than in TDP-43^A315T^ mice (*p* = 0.03), suggesting that a more even distribution of taxa can be found in WT controls compared to TDP-43^A315T^ mice. The Venn diagram exhibited the exact number of ASVs common for the four groups (174). This number was higher than the number of unique ASVs in WT controls exposed to O_3_, but lower than the unique ASVs observed in the other groups (Fig. 5B). Actually, the gut microbiome composition of TDP-43^A315T^ mice and WT controls yielded significantly different compositions (Fig. 6). TDP-43^A315T^ mice showed a significant increase in ASVs within Proteobacteria phyla like *Parasutterella* and *Escherichia*/*Shigella* genus. In addition, several ASVs within specific genera were observed in TDP-43^A315T^ mice but not in WT controls (e.g. *Ruminococcus*, *Parabacteroides*, *Marvinbryantia*, *Blautia*) (Supplementary Fig. S3).

### O_3_ exposure did not impair the NMJs in the skeletal muscle of TDP-43^A315T^ mice

**Figure 7:**
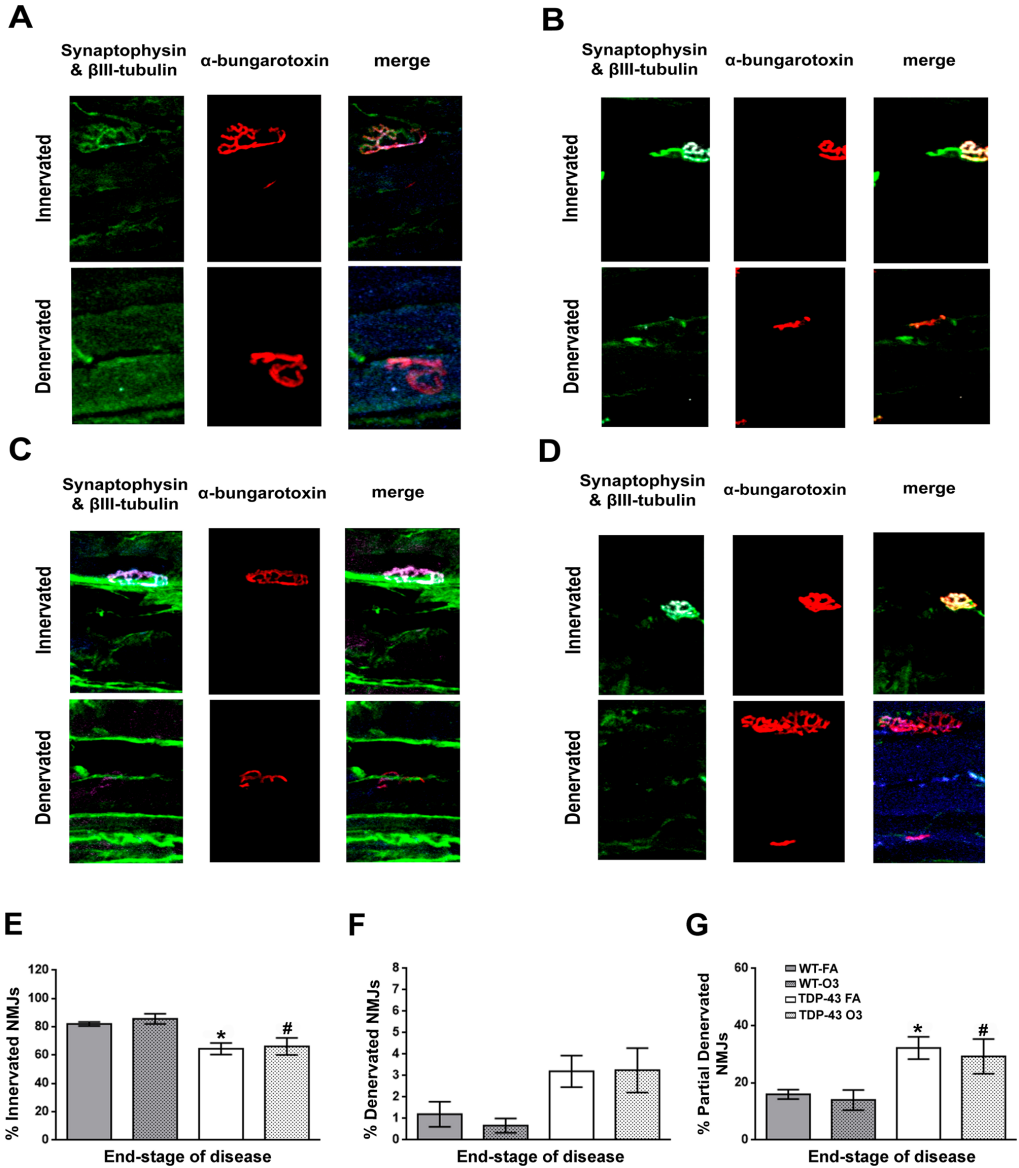
**O**_**3**_ **exposure did not alter the NMJs denervation state in the TA muscle in TDP-43**^**A315T**^ **mice at the end-stage of the disease.** Representative examples of NMJ immunostaining in TA muscle of WT mice exposed to FA (n = 6 mice) (**A**) or O_3_ (n = 8 mice) (**B**), and TDP-43^A315T^ mice exposed to FA (n = 5 mice) (**C**) or O_3_ (n = 7 mice) (**D**). Quantification of innervation (**E**), denervation (**F**) and partial denervation (**G**) at the end-stage of disease. Values are expressed as the mean ± SEM for the different groups. Comparison between groups was performed by two-way ANOVA followed by Dunnett’s post hoc test to compare all groups with WT-FA, while Tukey’s post hoc test was used for multiple comparisons between all groups, where * *p* < 0.05 vs. WT mice FA-exposed; # *p* < 0.05 vs. WT mice O_3_-exposed. Abbreviations: WT, Wild-type mic; TDP-43, TDP-43^A315T^ mice; FA, filtered air; O_3,_ ozone.

We next analised alterations in NMJs in TDP-43^A315T^ mice compared to age-matched WT littermate controls in responses to FA or O_3_ exposure, as the hypothalamus regulates skeletal muscle metabolism^47^, and NMJ pathology is well-known in mouse models of ALS^48^. To this aim we examined the TA muscle by IHC analysis (Fig. 7). Densitometric analysis demonstrated a significant effect of genotype on the percentage of innervated and denervated (*p* < 0.0001, respectively) NMJs in the TA muscle of TDP-43^A315T^ mice compared to WT (Fig. 7E–G). Tukey’s post hoc test demonstrated a significant decrease on the percentage of innervated NMJs in the TA muscle of TDP-43^A315T^ mice exposed to FA and O_3_ relative to WT mice in response to FA (*p* = 0.0013 and *p* = 0.007, respectively) and O_3_ exposure (*p* = 0.0007 and *p* = 0.003, respectively) (Fig. 7E). Similarly, IHC analysis showed a significant decrease on the percentage of denervated NMJs in the TA muscle of TDP-43^A315T^ mice exposed to FA and O_3_ compared to WT in response to FA and O_3_ exposure (*p* = 0.002 in all cases; Fig. 7F). There was a slight decrease in partially denervation of NMJs in the TA muscle of TDP-43^A315T^ mice O_3_-exposed compared to FA-exposed, however, the differences were not statistically significant (Fig. 7G).

## Discussion

Although the cellular basis for motor neuron degeneration in ALS is not yet fully understood, it is increasingly recognized that energy homeostasis (the balance between energy intake and expenditure) appears to be compromised in ALS^49^. Indeed, ALS patients develop prominent changes in weight and eating behavior that result from and mediate the underlying neurodegenerative process. In this context, emerging research suggests that these alterations may be mediated through changes in the hypothalamic function^50^.

Hypothalamic neuropeptides derived from POMC provide a strong anorexigenic effect (i.e. decreases food intake), while NPY/AgRP neurons have a potent orexigenic effect (i.e. increase food intake). Here, we report a downregulation of *AgRP* mRNA levels and a significantly upregulation of *POMC* transcripts in TDP-43^A315T^ mice compared to WT, which is in accordance with previously data reporting a progressive loss of body weight in TDP-43^A315T^ mice^13,51–54^. In addition, although no statistical difference was obtained, our results show a reduction of NPY protein expression levels in TDP-43^A315T^ mice O_3_-exposed relative to FA-exposed. It is conceivable that changes in the expression of NPY in the hypothalamus of TDP-43^A315T^ mice could potentially be due to modifications in food intake. Indeed, in a situation of negative energy balance, such as the malnutrition occurring in the majority of ALS patients^55,56^, the expression of NPY would be normally increased^57,58^. However, future experiments with a larger sample size should try to corroborate this hypothesis.

Besides sensitive genes involved in metabolism, we also examined leptin signaling in the hypothalamus of TDP-43^A315T^ mice, as central hypothalamic leptin signaling has a critical role in promoting energy homeostasis via modulation of food intake and energy expenditure^59^. Although the precise dynamics of Ob-Rb regulation in the hypothalamus is not completely understood, no differences in Ob-Rb protein expression levels between groups were obtained. However, a significant decrease in serine phosphorylation of Akt was determined in the hypothalamus of TDP-43^A315T^ mice in response to O_3_ exposures. The alterations in Akt levels could partly explain the hypoglycemic state observed in TDP-43^A315T^ mice^13^, as Akt is an important target in the regulation of glucose and energy metabolism^38^, which is in accordance with previous data from our group showing how O_3_ exposure resulted in higher plasma glucose levels at later time points^13^. In addition, this data might reflect disruption of insulin signaling in the hypothalamus of TDP-43^A315T^ mice, as Akt signaling pathway is part of the insulin cascade^60^. Indeed, insulin signaling in the hypothalamus plays a role in maintaining body weight^61^, and insulin resistance is related to disease severity in ALS^62^. However, future experiments should try to corroborate this hypothesis.

Considering that BAT has profound effects on body weight and metabolism in rodents^63^, it is conceivable that the reported improvement on loss of body weight in TDP-43^A315T^ mice in response to O_3_ exposure, could be due to the effect of this gas to modify its thermogenic function. Indeed, the ability of this adipose tissue to expend energy has increased interest in stimulating thermogenesis to treat metabolic diseases such as obesity and diabetes type II^64^. In this context, our results confirm a significant upregulation of genes involved in brown adipocyte differentiation, including *AdipoQ*, *Fabp4*, *Prdm16* and *PPARγ* mRNAs in BAT of TDP-43^A315T^ mice in response to FA compared with O_3_ exposure. We also found significant effects of O_3_ exposure on the expression profile of genes involved in BAT-mediated thermogenesis such as *UCP1* and *PGC1α*. These data are of interest because evidence supports that the decrease in thermogenesis is likely associated with a greater predisposition to body weight gain^65^, which is in accordance with previous data from our group showing a less severe decline disease-associated weight loss in TDP-43^A315T^ mice exposed to O_3_. Indeed, we also found a significant upregulation of *GLUT4* mRNA levels in the BAT of TDP-43^A315T^ mice in responses to O_3_ exposure, which is an interesting data as we previously reported plasma glucose levels were highest at the end-stage of disease after O_3_ exposure in TDP-43^A315T^ mice^13^. Finally, concomitantly to the decrease in the expression profile of *AdipoQ*, *Fabp4*, *Prdm16*, *PPARγ, UCP1* and *PGC1α *in the BAT of TDP-43^A315T^ mice, our results confirm a significant downregulation of *Ob-Rb* mRNA expression levels in responses to O_3_ exposure, which is in accordance with experimental data showing how obese mice that lack of Ob-Rb receptor showed a decreased BAT thermogenic capacity^66^. As a whole, these transcriptional modifications in TDP-43^A315T^ mice exposed to O_3_ might reflect the physiological response of the hypothalamus to this gas to overcome adipose atrophy and loss of body weight. Indeed, growing experimental research suggests the importance of the hypothalamus and the role of hypothalamic peptides and neurons in the control of BAT thermogenesis^67^. However, future experiments should try to corroborate this hypothesis.

In attempt to have a better knowledge on the potential major role of O_3_ exposure to induce controlled metabolic effects, we examined fecal microbiome composition in TDP-43^A315T^ mice compared to WT. Gut microbiome has been implicated in ALS development^68^, and thus, it is conceivable that the metabolic changes determined in TDP-43^A315T^ mice in responses to O_3_ exposure could be also due to a compensatory effect of O_3_ on gut dysbiosis (an imbalance in the gut microbiota community). Indeed, in ALS disease gut dysbiosis affects the central nervous system via pro-inflammatory mediators (i.e. cytokines and hormone-like molecules), thus, impacting gut-brain communications^69^. In this context, our data showed how the gut microbiome composition of TDP-43^A315T^ mice yielded significantly different composition compared with WT controls. Although no statistical changes were found on gut bacterial communities in TDP-43^A315T^ mice in response to O_3_ compared with FA exposure, an increase in the relative abundance of genera like *Bacteroides*, *Parabacteroides* or *Marvinbryantia* was observed as a result of such exposure (Supplementary Fig. S1). In addition, gut microbiome composition of WT mice showed a significant increase in *Lachnospiraceae* NK4A136 group as a result of O_3_ exposure. This data is of interest because this group is a potential butyrate-producer associated with probiotic activity in mice^70^. Indeed, it has been found that butyrate is one of the main short-chain fatty acids (SCFAs) produced by microbiota helping to maintain gut barrier integrity and inhibit inflammation^71^. These results might indicate the capacity of this O_3_ to restore physiological homeostasis and potentially improve dysbiosis.

Finally, as metabolic alterations in ALS are associated with the progression of disease pathology, we examined potential changes in NMJ innervation in TA muscles of TDP-43^A315T^ mice, as degenerative processes in the skeletal muscle, particularly involving NMJs, are observed throughout disease progression in ALS^72,73^. Our densitometric analysis showed no significant changes on NMJs in TA muscles in TDP-43^A315T^ mice in response to O_3_ exposure compared with FA exposure, although a significant impairment of NMJs in TDP-43^A315T^ mice compared to WT controls was found, which is in agreement with previous data reported in mutant TDP-43 mice (i.e. Tg NEFH-hTDP-43ΔNLS or TDP-43 rNLS bigenic mice) showing a progressive NMJs denervation followed by spinal cord motor neuron loss^74^.

In summary, our study provides the first experimental evidence underlying the potential effect of O_3_ exposure on hypothalamic function in TDP-43^A315T^ mice, which might result in modifications on neural signaling, affecting thermogenesis and energy homeostasis in ALS. Molecular biology analysis has demonstrated that O_3_ modified the expression profile of hypothalamic neuropeptides, and significantly altering phosphorylation levels of Akt, concomitantly to decrease the expression of genes involved in metabolism and thermogenesis in the BAT of TDP-43^A315T^ mice O_3_-exposed. Composition of fecal gut microbiome of O_3_-exposed TDP-43^A315T^ mice varied significantly compared to WT controls, and densitometric analysis of NMJs, indicated that O_3_ does not impair the progression of disease in the skeletal muscle. However, further functional studies are necessary to determine the mechanism of actions of O_3_ which may provide a new avenue for therapeutic development for this fatal condition.

## Supplementary Information

Supplementary Figure 1.

Supplementary Figure 2.

Supplementary Figure 3.

## Data Availability

The datasets generated and/or analysed during the current study are available upon reasonable request to the corresponding author, Dr Carmen M. Fernandez-Martos (cmfernandezm@sescam.jccm.es; Carmen.fernandez-martos@utas.edu.au), and the raw demultiplexed sequence data (amplicon reads) have been uploaded to the Sequence Read Archive (SRA) with BioProject accession number PRJNA886986 (https://www.ncbi.nlm.nih.gov/bioproject/886986). Additional material including source data is available online.
