# Supplementary material for: Ozone modified hypothalamic signaling enhancing thermogenesis in the TDP-43A315T transgenic model of Amyotrophic Lateral Sclerosis
Source: Sci Rep. 2022 Dec 2;12:20814. doi: 10.1038/s41598-022-25033-4 (PMC9718766; doi:10.1038/s41598-022-25033-4)
Supplement: Supplementary file 1 — Supplementary Figure 1. [file 41598_2022_25033_MOESM1_ESM.docx]

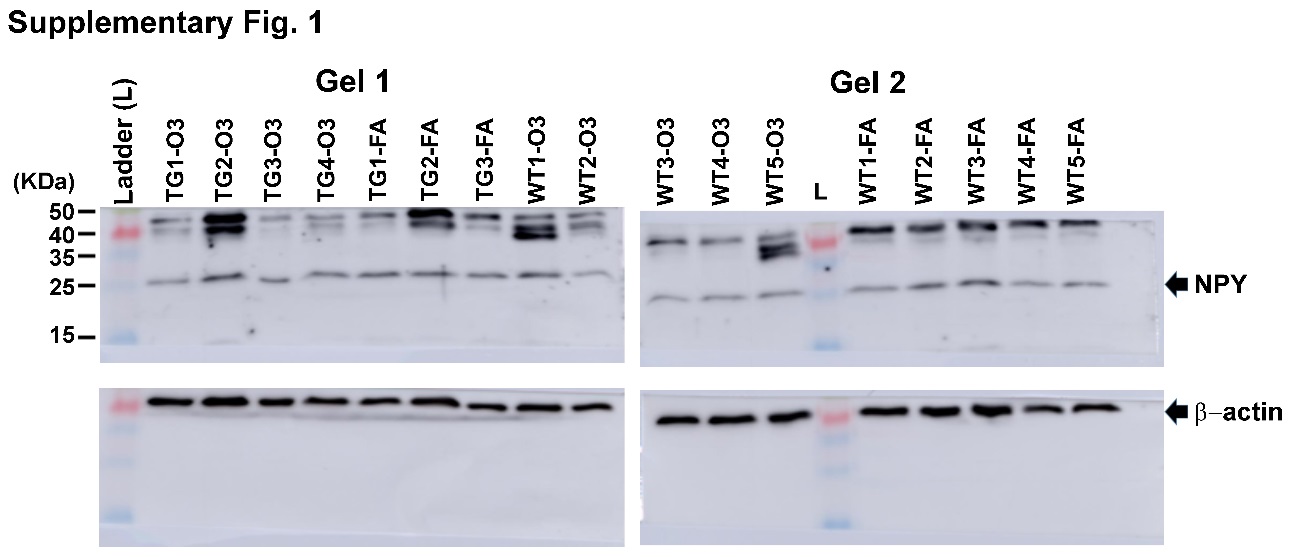
**Suppl. Fig. S1. Blots of Figs 1C**. The blotted membranes (gel 1 and 2) were trimmed to the expected molecular weight range and staining with NPY antibody, then the antibody was stripped, and the trimmed membranes were re-probed with anti-β-actin antibody. Abbreviations: WT, Wild-type mice; TG, TDP-43^A315T^ mice; FA, filtered air; O_3_, ozone.
