# Supplementary material for: Ozone modified hypothalamic signaling enhancing thermogenesis in the TDP-43A315T transgenic model of Amyotrophic Lateral Sclerosis
Source: Sci Rep. 2022 Dec 2;12:20814. doi: 10.1038/s41598-022-25033-4 (PMC9718766; doi:10.1038/s41598-022-25033-4)
Supplement: Supplementary file 2 — Supplementary Figure 2. [file 41598_2022_25033_MOESM2_ESM.docx]

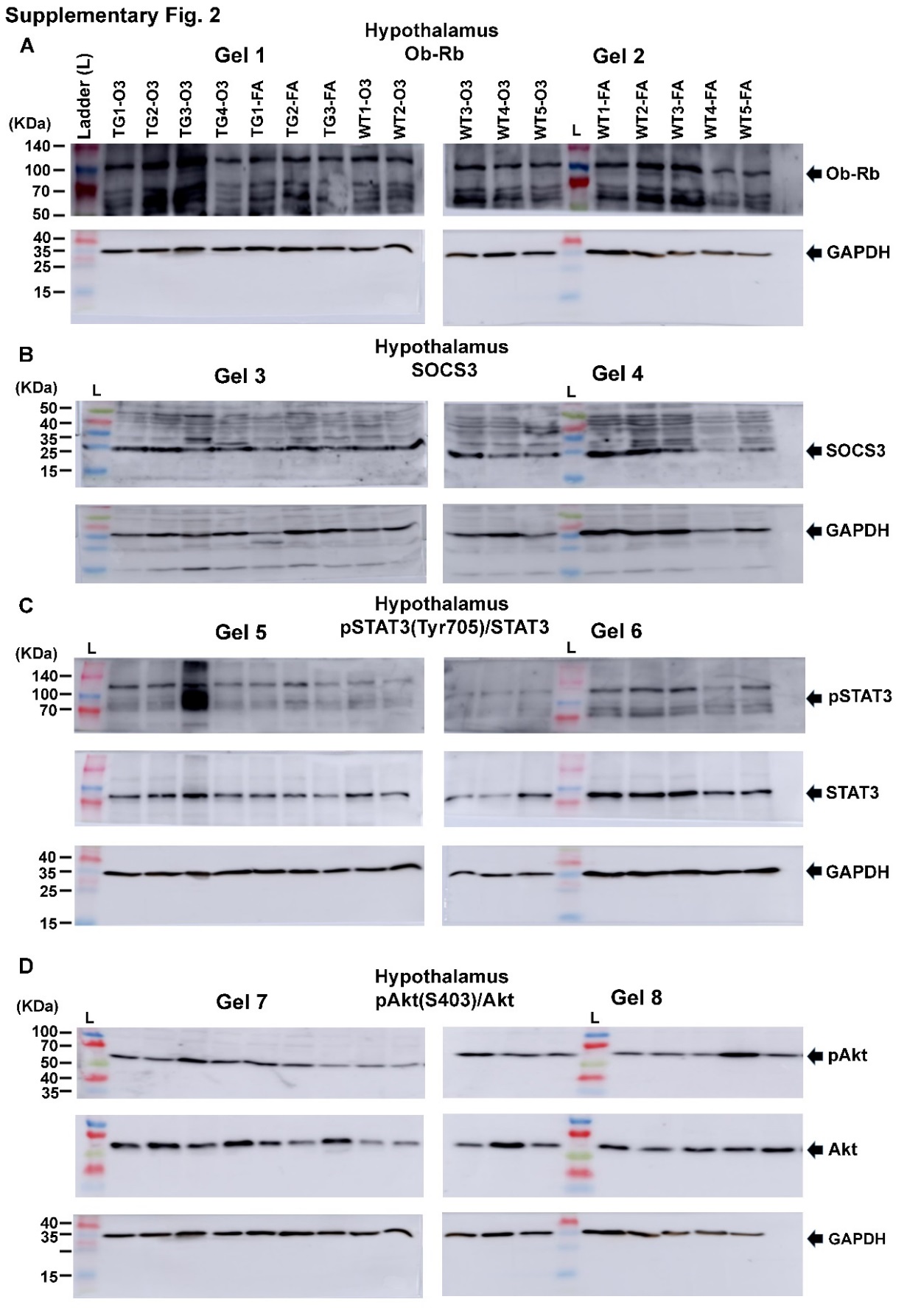


**Suppl. Fig. S2. Blots of Figs 2A, 2B, 2C and 2D**. The blotted membranes (gels 1 to 8) were trimmed to the expected molecular weight range and staining with (2A) Ob-Rb and (2B) SOCS3, then the antibody was stripped, and the trimmed membranes were re-probed with anti-GAPDH antibody, respectively; and with (2C) pSTAT3(Tyr705) and (2D) pAkt(S405), then the total anti-Akt, respectively.
