# Supplementary material for: Ozone modified hypothalamic signaling enhancing thermogenesis in the TDP-43A315T transgenic model of Amyotrophic Lateral Sclerosis
Source: Sci Rep. 2022 Dec 2;12:20814. doi: 10.1038/s41598-022-25033-4 (PMC9718766; doi:10.1038/s41598-022-25033-4)
Supplement: Supplementary file 3 — Supplementary Figure 3. [file 41598_2022_25033_MOESM3_ESM.docx]

**Supplementary Fig. 3**


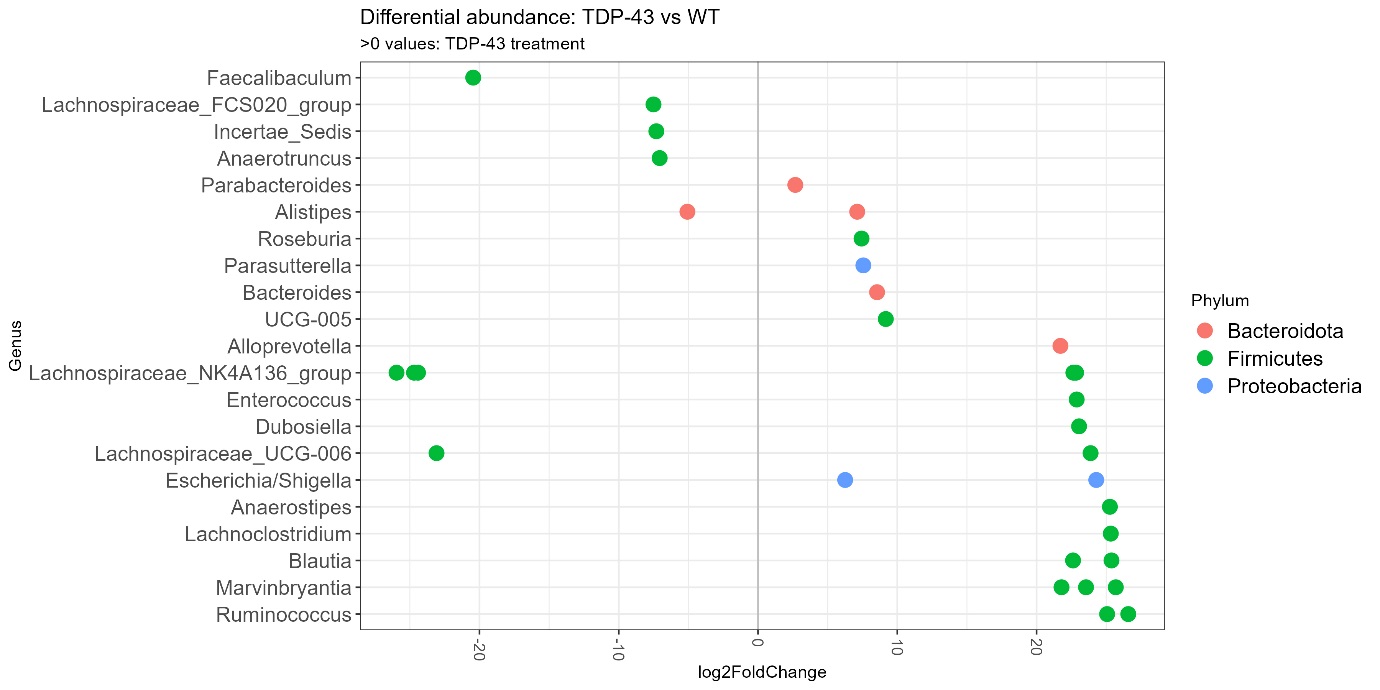


**Suppl. Fig. S3. Relative abundance of the most prevalent bacterial phyla in TDP-43^A315T^ *vs.* WT mice.** Log2 fold-change analysis of the ASVs obtained from the TDP-43^A315T^ mice compared to WT controls in response to FA or O_3_ exposures at the end-stage of disease. Bacterial ASVs grouped by genus and coloured by phylum (legends). Log2 fold-change values indicate the strength and direction of the association to WT (<0) and TDP-43 (>0) mice. Genera observed in this figure were statistically significantly different between locations at *p*<0.01 corrected. Abbreviations: WT, Wild-type mice; TG, TDP-43, TDP-43^A315T^ mice; FA, filtered air; O_3_, ozone.
